# The Role of Persistent Organic Pollutants in Obesity: A Review of Laboratory and Epidemiological Studies

**DOI:** 10.3390/toxics10020065

**Published:** 2022-02-02

**Authors:** Jan Aaseth, Dragana Javorac, Aleksandra Buha Djordjevic, Zorica Bulat, Anatoly V. Skalny, Irina P. Zaitseva, Michael Aschner, Alexey A. Tinkov

**Affiliations:** 1 Research Department, Innlandet Hospital Trust, P.O. Box 104, 2381 Brumunddal, Norway; 2 Faculty of Health and Social Sciences, Inland Norway University of Applied Sciences, P.O. Box 400, 2418 Elverum, Norway; 3 Department of Toxicology “Akademik Danilo Soldatović”, University of Belgrade-Faculty of Pharmacy, Vojvode Stepe 450, 11000 Belgrade, Serbia; dragana.javorac@pharmacy.bg.ac.rs (D.J.); aleksandra.buha@pharmacy.bg.ac.rs (A.B.D.); zorica.bulat@pharmacy.bg.ac.rs (Z.B.); 4 World-Class Research Center “Digital Biodesign and Personalized Healthcare”, IM Sechenov First Moscow State Medical University (Sechenov University), 119435 Moscow, Russia; skalny3@microelements.ru; 5 Department of Bioelementology, KG Razumovsky Moscow State University of Technologies and Management, 109004 Moscow, Russia; 6 Laboratory of Ecobiomonitoring and Quality Control, Yaroslavl State University, 150003 Yaroslavl, Russia; irisha-zip@yandex.ru; 7 Department of Molecular Pharmacology, Albert Einstein College of Medicine, Bronx, NY 10461, USA; michael.aschner@einsteinmed.org; 8 Laboratory of Molecular Dietetics, IM Sechenov First Moscow State Medical University (Sechenov University), 119435 Moscow, Russia

**Keywords:** obesity, dioxin, polybromated diphenylethers, dichlorodiphenyltrichloroethane, polychlorinated biphenyls, polyaromatic hydrocarbons, bisphenol A, phthalates, perfluorinated compounds, adipogenesis

## Abstract

Persistent organic pollutants (POPs) are considered as potential obesogens that may affect adipose tissue development and functioning, thus promoting obesity. However, various POPs may have different mechanisms of action. The objective of the present review is to discuss the key mechanisms linking exposure to POPs to adipose tissue dysfunction and obesity. Laboratory data clearly demonstrate that the mechanisms associated with the interference of exposure to POPs with obesity include: (a) dysregulation of adipogenesis regulators (PPARγ and C/EBPα); (b) affinity and binding to nuclear receptors; (c) epigenetic effects; and/or (d) proinflammatory activity. Although in vivo data are generally corroborative of the in vitro results, studies in living organisms have shown that the impact of POPs on adipogenesis is affected by biological factors such as sex, age, and period of exposure. Epidemiological data demonstrate a significant association between exposure to POPs and obesity and obesity-associated metabolic disturbances (e.g., type 2 diabetes mellitus and metabolic syndrome), although the existing data are considered insufficient. In conclusion, both laboratory and epidemiological data underline the significant role of POPs as environmental obesogens. However, further studies are required to better characterize both the mechanisms and the dose/concentration-response effects of exposure to POPs in the development of obesity and other metabolic diseases.

## 1. Introduction

Obesity is considered as a worldwide epidemic posing significant negative health effects. Specifically, current estimates indicate that more than 1.9 billion adults are overweight, with more than 650 million of them characterized as obese [1]. It has also been extrapolated that the majority of the global adult population will be overweight/obese by 2030 [1]. In addition to adults, the prevalence of obesity in children is also characterized by a drastic increase, reaching up to 5.6% and 7.8% in boys and girls, respectively [2]. Of note, parental obesity is associated with a higher risk and severity of obesity in children [3].

Obesity leads to significant health effects [4] due to association with metabolic disturbances, including insulin resistance and type 2 diabetes mellitus, atherogenic dyslipidemia, and hypertension, altogether clustered to the metabolic syndrome [5] being the major cardiovascular risk factor [6]. Obesity is also associated with other systemic disorders including respiratory [7] and neurological [8] diseases, as well as cancer [9]. Finally, obesity is considered one of the key risk factors for COVID-19 severity and mortality [10].

Beyond the key role of caloric excess in the development of obesity, multiple biological, environmental, and behavioral factors were shown to affect susceptibility to obesity [11]. The potential role of exposure to chemical toxins in expanding the obesity epidemic was proposed in 2002 [12]. Further studies have shown a significant association between exposure to persistent organic pollutants (POPs) and obesity in humans [13], although certain inconsistencies in the epidemiologic data exist [14].

POPs refer to a chemically heterogeneous group of pollutants that are categorized according to their origin to (i) intentionally produced POPs including organochlorine pesticides and industrial chemicals (polychlorinated biphenyls), and (ii) unintentionally produced agents including polyaromatic hydrocarbons, dioxins, and furans [15,16].

POPs are chemically resistant to environmental degradation and hence persist for long periods of time in the environment and can accumulate and pass from one species to the next through the food chain [17]. Some POPs have been banned or have been nominated to be banned from production at the Stockholm Convention due to their persistency in the environment and their bio-accumulative and toxic properties in both animals and humans. Those chemicals which are still on the market are regulated through the Registration, Evaluation, and Authorization of Chemicals (REACH). Nevertheless, POPs are still to be found in both human and animal tissues worldwide [18,19,20].

These chemicals also accumulate in human tissue with long biological half-lives; they can cross the placenta during gestation. The main route of exposure to these chemicals is through dietary intake, while the environmental exposure pathways, mainly dermal exposure, account for less than 2% of exposure for most of them [21].

Being hydrophobic, brominated and chlorinated POPs accumulate predominantly in lipid rich tissues [22], while perfluorinated compounds have a higher affinity for plasma proteins [23]. Various POPs chemicals are present in children’s blood and breast milk, and due to their ability to cross both the placental and the blood brain barriers, can reach the developing nervous system [24]. However, adipose tissue is the one that plays a major role in the storage and toxicokinetics of POPs. La Merrill et al. [25] suggested some additional functions of adipose tissue in context of POPs toxicity. Adipose tissue acts as a unique buffering system by protecting other organs and tissue by sequestering POPs. However, at the same time, this tissue represents a continual source of internal exposure to POPs. 

POPs were shown to promote obesogenic effects through endocrine disruption, thus being considered as obesogens [26,27]. Obesogens have been shown to affect adipose tissue development and functioning through interference with key adipocyte metabolic pathways, including the regulation of adipogenesis [28]. Such dysregulation may also be mediated by the epigenetic effects of the pollutants [29]. In addition, alterations in other endocrine tissues, as well as hypothalamic centers, may contribute to energy dyshomeostasis upon exposure to POPs [30]. Moreover, it has been observed that accumulation of POPs in adipocytes promotes inflammation in adipose tissue, thus contributing to the dysfunction of the tissue [31]. However, the mechanisms of the obesogenic effects of various POPs may be quite different [31] and have yet to be fully characterized. 

Given the above observations, the objective of the present review is to discuss the key mechanisms linking exposure to POPs to adipose tissue dysfunction and obesity in laboratory studies, as well as to review the epidemiological evidence supporting this association. 

## 2. Perfluorinated Compounds

**Figure 1:**
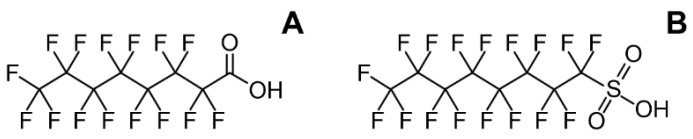
The chemical structures of perfluorooctanoic acid (PFOA) (**A**) and perfluorooctane sulphonate (PFOS) (**B**).

The role of perfluorooctanoic acid (PFOA) (Figure 1A) and perfluorooctane sulphonate (PFOS) (Figure 1B) in obesity has yet to be fully clarified, although some data derived from epidemiological studies suggest that PFOS and PFOA exposures are associated with overweight and obesity [32]. Higher levels of PFOS and PFOA were found in mothers with obesity and in underweight mothers when compared to those of a normal weight [33]. Moreover, prenatal exposure to PFAS has been linked with obesity, metabolic disorders, and alterations in children’s growth [34,35,36]. In a large multicenter prospective cohort study, the “European Youth Heart Study” performed in young participants (N = 369), scientists found that PFOS and PFOA exposure predicted adiposity at 15 and 21 years of age [37]. A recently published cross-sectional study in US children from 12–18 years of age (N = 2473) showed a dose-dependent association between obesity and PFAS exposure [38]. Nonetheless, the European Food Safety Authority reported in 2020 that there is insufficient data to support a link between PFAS exposure and obesity, thus the explanation for this association requires further research [39]. 

In contrast, laboratory data demonstrate that perfluorinated compounds are potent inducers of adipogenesis through interference with peroxisome proliferator-activated receptor gamma (PPARγ) signaling and other pathways, being most prominent for sulfonated perfluoroalkyl acids, whereas carboxylated agents showed lesser alterations of mouse 3T3-L1 cells gene expression [40]. A similar mechanism was responsible for the PFOA-induced inhibition of human mesenchymal stem cells (MSC) osteogenic differentiation and adipogenesis stimulation [41]. In addition to PPARγ binding and the associated up-regulation of adipogenesis [42], PFOA was found to increase PPARγ transcription and the demethylation of PPARγ promoters during 3T3-L1 preadipocyte differentiation [43]. A study in the nematode *C. elegans* also demonstrated the involvement of PPAR, mitogen activated protein kinase (MAPK), and transforming growth factor beta (TGFβ) signaling in the obesogenic effect of PFOA [44]. At the same time, an in vivo study of perinatal PFOA exposure demonstrated a sex-specific response with more profound metabolic alterations in female C57BL/6JxFVB mice [45]. 

The observed adipogenic effects were accompanied by an increase in insulin-stimulated glucose uptake through the up-regulation of glucose transporter type 4 (GluT4) and insulin receptor substrate 1 (IRS1) expression in murine 3T3-L1 preadipocytes [46]. However, this observation contrasts with findings from an in vivo study demonstrating PFOA-induced insulin resistance in exposed Balb/c mice. These effects are proposed to be mediated by the down-regulation of protein kinase B (Akt) mRNA expression and phosphorylation, as well as increased phosphatase and tensin homolog (PTEN) mRNA expression and protein levels [47]. 

Similarly, PFOA and PFOS were shown to decrease osteopontin, osteonectin, osteocalcin, and β-catenin expression in human bone marrow-derived mesenchymal stem cells (hBMSCs), thus being indicative of reduced osteogenesis, whereas the expression of adipogenesis-specific marker genes PPARγ, CCAAT/enhancer-binding protein alpha (C/EBPα), lipoprotein lipase (LPL), and leptin were up-regulated [41]. It has been also demonstrated that in parallel with induction of PPARγ and C/EBPα expression, PFOS-induced adipogenesis was associated with the activation of the nuclear factor-erythroid factor 2-related factor 2 (Nrf2) pathway in murine 3T3-L1 preadipocytes [46]. In addition to PPARγ, proadipogenic effects of PFOS may involve induction of activating protein 2 (ap2) [48], as well as PPARα and PPARβ mRNA expression, affecting stem cell differentiation in hBMSCs [49]. The modulation of DNA methylation may also be considered a potential mechanism of the impact of PFOS on adipogenesis [50].

An adipogenic effect was also demonstrated for certain other perfluorinated compounds. Specifically, perfluorobutanesulfonic acid (PFBS) used as a substituent for perfluorooctanesulfonic acid (PFOA) was demonstrated to be a proadipogenic agent, promoting the differentiation of 3T3-L1 preadipocytes to adipocytes by up-regulating PPARγ and C/EBPα transcription factors and lipogenic acetyl-CoA carboxylase (ACC) and fatty acid synthase (FAS) [51]. In comparison to PFOS, chlorinated polyfluorinated ether sulfonates (Cl-PFAESs) were shown to be more potent stimulators of 3T3-L1 adipogenesis through the PPARγ pathway [52]. Perfluorinated alkyl acids (PFAAs) are also capable of inducing adipogenesis in 3T3-L1 cells at human blood-based exposure levels [53].

**Figure 2:**
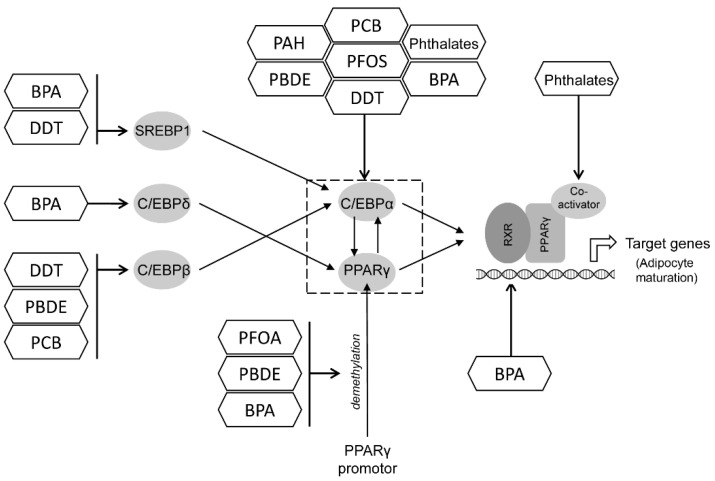
The potential mechanisms underlying the adipogenic effects of POPs in adipocytes. Briefly, POPs were shown to up-regulate PPARγ and C/EBPα signaling through a variety of mechanisms, including the activation of C/EBPδ and C/EBPβ and SREBP1, as well as an increase in PPARγ promotor demethylation. The activation of PPARγ results in the up-regulation of adipocyte-specific gene expression and adipocyte maturation. BPA—bisphenol A; PCB—polychlorinated bipenyls; PBDE—polybrominated diphenyl esters; PAH—polyaromatic hydrocarbons; PFOS—perfluorooctane sulphonate; PFOA—perfluorooctanoic acid; DDT—dichlorodiphenyltrichloroethane; DES—diethylstilbestrol; SREBP1—sterol regulatory element-binding protein 1; C/EBP—CCAAT-enhancer-binding proteins; PPARγ—peroxisome proliferator-activated receptor gamma; RXR—retinoid X receptor.

Altogether, the existing data demonstrate that PFOS and/or PFOA exposure may promote adipogenesis through the up-regulation of PPARγ and C/EBPα signaling, thus contributing to an increased risk of obesity, although the epidemiological data have yet to confirm this association (Figure 2).

## 3. Polybrominated Diphenyl Ethers (PBDE)

**Figure 3:**
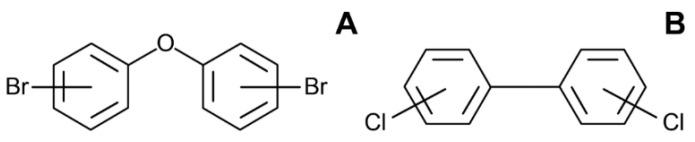
Common formulas of the persistent organic pollutants polybrominated diphenyl ethers (PBDE) (**A**) and polychlorinated biphenyls (PCB) (**B**).

Polybrominated diphenyl ethers (PBDEs) (Figure 3A) are man-made chemicals and environmental pollutants used in industry as flame retardants in various commercial goods [54]. PBDEs, due to their lipophilic properties, tend to accumulate in adipose tissue, possibly changing its function and raising the risk of metabolic diseases. Several studies suggested a link between PBDEs and obesity and metabolic syndrome [55,56].

In individuals with obesity, the accumulation of certain PBDEs in human adipose tissues is linked to insulin resistance [57]. In a study conducted of 224 mothers during pregnancy and afterwards, including their children at age 7, it was revealed that the levels of BDE 47 and BDE 153 in maternal blood were positively associated with the body mass index (BMI) of boys. Contrary, in girls, the PBDE levels in maternal blood negatively correlated with BMI [58]. Furthermore, the serum levels of BBDEs were found to be positively associated with the expression of obesity biomarkers in subcutaneous and visceral adipose tissue, such as leptin, adiponectin, tumor necrosis factor α (TNFα), and PPARγ, implying that exposure to these pollutants may contribute to the development of obesity in humans [59].

In agreement, laboratory data have shown that PBDE exposure potentiates dexamethasone-induced adipogenesis in 3T3-L1 cells with the up-regulation of C/EBPα, PPARγ, and liver X receptor alpha (LXRα) [60,61]. Specifically, PBDE 99 was shown to up-regulate C/EBPβ with subsequent the activation of C/EBPα and PPARγ along with promotion of mitotic clonal expansion. In addition, the methylation status of PPARγ promoter was found to be reduced in response to PBDE 99 exposure [62]. Furthermore, a stimulatory effect of BDE-47 on 3T3-L1 adipocyte differentiation was shown to be dependent on *Pparγ2* gene induction and *Pparγ2* promoter demethylation [63].

However, some other studies indicated that an obesogenic effect of PBDE may be mediated by other distinct mechanisms. Specifically, adipogenesis stimulation by PBDE-47 may be dependent on the activation of purine metabolism, oxidative stress, and mitochondrial respiration [64]. These effects are also associated with a reduction of lipid catabolism through the inhibition of β-oxidation and increased lipid biosynthesis, altogether resulting in increased lipid accumulation [65], as well as the inflammatory infiltration of adipose tissue in C57BL/6J mice [66].

The systemic effects of PBDE may also significantly contribute to its modulation of metabolic risk in obesity. It has been proposed that the obesogenic effect of PBDE exposure may be related to reduced T4 levels with a subsequent decrease in systemic thyroid hormone effects in Wistar rats [67]. In addition, PBDEs also significantly alter the murine gut microbiome, which may, at least partially, mediate the association between PBDE exposure and metabolic syndrome [68].

In corroboration with epidemiological studies demonstrating the association between PBDE exposure and obesity, as well as the accumulation of PBDE in adipose tissue, laboratory data indicate that adipogenic effect of PBDE is mediated by the up-regulation of PPARγ and C/EBPα signaling (Figure 1) through a variety of mechanisms including epigenetic effects, modulation of oxidative stress, adipose tissue inflammation, thyroid functioning, and gut microbiota.

## 4. Dichlorodiphenyltrichloroethane (DDT)

**Figure 4:**
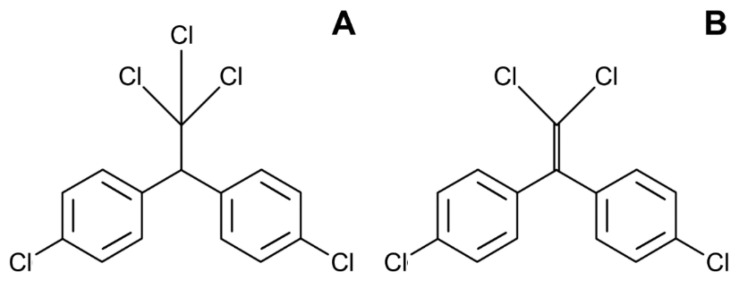
The structures of dichlorodiphenyltrichloroethane (DDT) (**A**) and its toxic metabolite dichlorodiphenyldichloroethane (DDE) (**B**).

Introduced as an insecticide in the 1940s, dichlorodiphenyltrichloroethane (DDT) (Figure 4A) is a persistent organic pollutant and endocrine disrupting chemical. In the last decade, a huge number of studies aimed to evaluate the association of the serum and fat levels of DDT and its metabolite dichlorodiphenyldichloroethylene (DDE) (Figure 4B) with body weight, visceral obesity, or BMI [69,70]. 

In a study conducted in Spain on 298 participants, a positive association was found between lipid DDT levels and BMI [71]. A larger study encompassing 775 men and 808 women has shown a positive correlation between BMI and DDT serum levels [72]. Interestingly, a similar trend was observed in children. The positive connection between DDE/DDT prenatal exposure and childhood BMI and growth patterns was determined in the CHAMACOS cohort study, which included 249 participants. The study showed that this correlation was stronger in boys than in girls [73,74,75,76]. Certain studies evaluated the association between obesity and prenatal DDT exposure. The Child Health and Development Studies in California demonstrated the effects of in utero exposure to DDT on middle-aged daughters (44–53 years, N = 511). Researchers have found a higher risk for being overweight (26–31%) as estimated from the BMI and waist circumference in the DDT exposed group [77]. In addition, in utero exposure to DDT was found to be associated with higher body weight in the postnatal period during the first and third years of life [78]. 

Moreover, DDT also plays a role in obesity-associated diseases. In a recent study, Henríquez-Hernández et al. have shown that DDT and/or its metabolites can contribute to obesity development and related diseases as recorded by altered fasting blood glucose and metabolic disorders [79]. Furthermore, adipose tissue DDT levels, a measure of the deposition of this chemical, were found to be positively associated with triglycerides, glycemic parameters, and central obesity in a study involving 100 participants [80].

Corroborating the epidemiological studies, both laboratory in vitro and in vivo studies revealed the potent adipogenic effects of DDT and DDE. Specifically, both substances were shown to promote adipogenesis in 3T3-L1 adipocytes, resulting in increased fatty acid synthase (FAS) and acetyl-CoA carboxylase (ACC) expression as well as lipid accumulation, likely mediated by the induction of C/EBPα and PPARγ expression. It has also been shown that both DDT and DDE are capable of up-regulating AMP-activated protein kinase-α (AMPKα) expression while reducing its phosphorylation [81]. These findings are in agreement with the observations in two adipocyte lines, 3T3-L1 and 3T3-F442A, that demonstrated a DDT-induced increase in C/EBPα and PPARγ protein levels, as well as C/EBPα interaction with DNA, absent influence on C/EBPβ expression [82]. 

The adipogenic effect of DDE was shown to be associated with the up-regulation of PPARγ expression, modulation of lipid metabolism, and a concomitant reduction of pluripotency genes in human adipose derived MSCs [83]. Correspondingly, DDT exposure reduced the self-renewal of human MSCs while increasing its adipogenic differentiation with increased mRNA expression of adipocyte-specific PPARγ, GluT4, and LPL. It is noteworthy that the effects of DDT were similar to those observed in response to estrogen exposure, while antiestrogen ICI 182780 ameliorated the influence of DDT, both being indicative of the role of estrogen receptor signaling in DDT-induced adipogenesis [84]. In a model of the suboptimal adipogenic differentiation of 3T3-L1 preadipocytes, DDE exposure increased lipid accumulation due to an increased expression of sterol regulatory element-binding protein 1 (SREBP1), fatty acid binding protein (FABP), and fatty acid synthase (FASN) [85].

It has been proposed that the effects of DDT exposure in adipocytes on the offspring from DDT-exposed rats may be significantly mediated by DNA methylation changes affecting adipogenesis regulation and metabolic functioning [86].

Of note, DDE was shown to induce insulin resistance in NIH3T3-L1 adipocytes through induction of adipose tissue endocrine dysfunction more efficiently than DDT [87]. In addition, perinatal DDT exposure in C57BL/6J mice may impair thermogenesis and reduce energy expenditure, thus promoting the dysregulation of lipid and carbohydrate metabolism and increasing susceptibility to metabolic syndrome [88].

**Figure 5:**
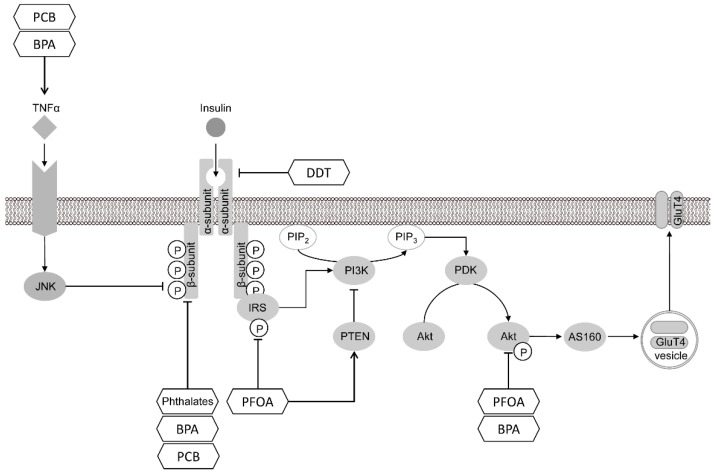
The molecular mechanisms involved in the dysregulation of insulin signaling in adipocytes upon exposure to POPs. POPs were shown to impair insulin signaling in adipocytes through the inhibition of insulin receptors and IRS phosphorylation, thus reducing downstream signaling from insulin receptors. Certain POPs may contribute to altered insulin signaling through the up-regulation of PTEN activity, as well as the inhibition of Akt phosphorylation. The proinflammatory effects of POPs may also contribute to the inhibition of insulin signaling and development of adipocyte insulin resistance. BPA—bisphenol A; PCB—polychlorinated bipenyls; PBDE—polybrominated diphenyl esters; PAH—polyaromatic hydrocarbons; PFOS—perfluorooctane sulphonate; PFOA—perfluorooctanoic acid; DDT—dichlorodiphenyltrichloroethane; DES—diethylstilbestrol; GluT4—glucose transporter type 4; JNK—c-Jun N-terminal kinases; AS160—Akt substrate of 160 kDa; PTEN—phosphatase and tensin homolog; IRS—insulin receptor substrate; PDK—phosphoinositide dependent kinase-1; PI3K—phosphoinositide 3-kinase; PIP3—phosphatidylinositol 3,4,5 trisphosphate; TNFα—tumor necrosis factor alpha.

Taken together, epidemiological studies provide strong evidence for the association between DDT exposure and the risk of obesity and obesity-related metabolic disturbances leading to metabolic syndrome. Such an obesogenic effect may be mediated by the up-regulation of C/EBPα and PPARγ signaling and a subsequent shift to adipogenesis (Figure 1), which is, at least in part, mediated by estrogen receptor signaling and epigenetic effects. The reduction of insulin sensitivity and energy expenditure may also contribute to the disturbances of carbohydrate and lipid metabolism upon DDT exposure (Figure 5).

## 5. Polychlorinated Biphenyls (PCBs)

Polychlorinated biphenyls (PCBs) (Figure 4B) are persistent organic chemicals that have been used in industry for more than 50 years. Although PCBs were globally banned in the late 1970s, they are still present in the environment. Exposure to PCBs has been shown to affect endocrine functions and metabolic processes by mimicking natural thyroid, estrogen, and androgen actions [89,90,91]. Recent published data suggest an association between exposure to PCBs and the development of obesity [92,93]. In the cross-sectional National Health and Nutrition Examination Survey’s (NHANES) 1999–2002 study of 721 non-diabetic adults with metabolic syndrome, the blood concentration of PCBs was linearly related to waist circumference [94]. Furthermore, in a longitudinal study with 12,313 participants, individuals with higher PCBs intake levels were at a greater risk of being obese, supporting the strong association between PCB exposure and the occurrence of obesity [95]. A number of studies have shown that the in utero exposure of embryos to PCBs can contribute to the development of metabolic syndrome, dyslipidemia, glucose intolerance, and other obesity-related metabolic disorders in postnatal life [55,96,97,98]. Maternal exposure to PCBs was associated with more pronounced obesity in girls than in boys, indicating a sex-dependent effect [99]. The overweight incidence in children has been shown to be sex-dependent in a study performed in 344 children, supporting an association of exposure to PCBs and overweight only in the girls’ group [97].

Based on findings from laboratory studies, PCBs have been implicated as endocrine-disrupting chemicals that are capable of disturbing lipid metabolism secondary to their accumulation in adipose tissue [100]. Specifically, PCB-118, PCB-153, and especially PCB-23 were shown to accumulate extensively within a lipid droplet of 3T3-L1 and mouse embryonic fibroblast-derived cultured adipocytes, being independent of caveolin 1 [101]. In turn, it has been proposed that molecular lipophilicity might be considered as a key factor in the regulation of PCB accumulation in adipose tissue [102]. 

PCBs were shown to impair adipogenesis through modulation of key adipogenesis regulators PPARγ and C/EPBs. Specifically, the adipogenic response to PCB-77 exposure was shown to be biphasic with increased 3T3-L1 adipocyte differentiation and PPARγ expression at low-dose treatments and inhibition upon high-dose exposure. Moreover, PCB-77 exposure in vivo was shown to induce obesity, dyslipidemia, and atherosclerosis in ApoE−/− mice [103]. In addition, PCB-153 was also shown to induce adipogenesis through PPARγ-independent mechanisms [104]. Aroclor 1254 (a mixture of chlorinated biphenyls) exposure in C57B6/J mice was shown to induce adipocyte differentiation via the stimulation of PPARγ signaling and the alteration of redox metabolism due to the inhibition of hepatocyte nuclear factor 1b (HNF1b) [105].

PCB 180 was shown to promote adipogenesis in murine 3T3-L1 preadipocytes and human visceral preadipocytes through the activation of C/EPBβ by reducing its SUMOylation and ubiquitination, thus reducing to hyperplastic obesity [106].

In agreement with PCBs’ ability to activate nuclear receptors [100], several effects of PCBs in adipose tissue, including insulin resistance, were shown to be largely attributed to the modulation of the aryl hydrocarbon receptor (AhR). Specifically, the administration of coplanar PCBs to C57BL/6 mice was shown to affect glucose homeostasis and insulin signaling through an AhR-mediated increase in adipocyte TNFα expression [107]. 

At the same time, an AhR-dependent increase in proinflammatory cytokine expression in human preadipocytes was found to be delayed in comparison to the classic AhR-targeted gene CYP1A1 [108]. Concomitantly, another study demonstrated that the PCB-153-induced glucose dyshomeostasis and inflammatory response may be mediated through NF-κB activation and the up-regulation of downstream proinflammatory cytokine genes in 3T3-L1 cells [109]. In addition to the up-regulation of adipocyte-specific proinflammatory cytokine production, PCB-138 is also capable of increasing 3T3-L1 adipocytes adipocyte resistance to TNFα-induced apoptosis via the up-regulation of surviving expression, thus promoting further lipid droplet enlargement assisted by Fsp27 and perilipin [110].

Another study demonstrated that the PCB-induced dysregulation of glucose metabolism in 3T3-L1 cells is reversed by resveratrol treatment and the resulting up-regulation of Nrf2 signaling, thus being indicative of the role of oxidative stress in PCB-associated insulin resistance [111]. 

The PCB-126-induced activation of AhR was also shown to be involved in the alteration of adipocyte beiging through the inhibition of UCP1 up-regulation in response to β-adrenergic stimulation, thus affecting energy metabolism in the subcutaneous human normal preadipocyte (NPAD) clone B cell line [112].

In addition, the activation of retinoid X and retinoic acid receptors (RAR), which play a significant role in adipogenesis regulation, may be also considered as a possible mechanism of the PCB adipogenic effect [113].

In vivo studies demonstrated that the hazardous effects of PCB exposure may be diet-dependent and aggravated in obesity. Specifically, in high-fat-fed mice, PCB-153 exposure resulted in a significant increase in body adiposity, hepatic steatosis, circulating leptin, adiponectin, resistin, and tPAI-1 concentrations, as well as the activation of hepatic lipid anabolism, whereas in STD-fed mice, no such effect was observed [114]. It is also notable that male zebrafish were more sensitive to PCB-induced alterations in lipid metabolism when compared to females, as evidenced by a higher expression of genes related to lipid biosynthesis and catabolism [115].

Generally, the existing data demonstrate that both life-long and prenatal PCB exposure may be considered as a risk factor for obesity due to the increased expression of adipogenic regulators PPARγ and C/EPBα, as well as C/EPBβ (Figure 2). In addition, PCB-induced AhR signaling was shown to be responsible for the development of adipose tissue inflammation, insulin resistance (Figure 5), and the inhibition of adipocyte energy expenditure and beiging.

## 6. Polycyclic Aromatic Hydrocarbons (PAHs)

**Figure 6:**
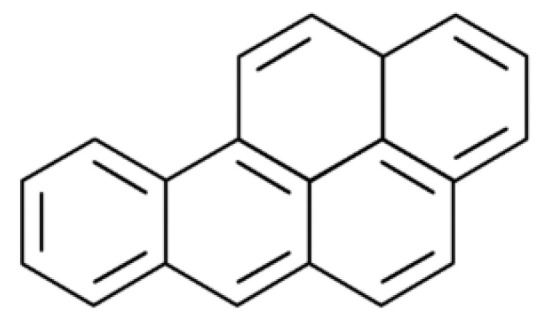
The structural formula of polycyclic aromatic hydrocarbon benzo[a]pyrene (BaP).

Polycyclic aromatic hydrocarbons (PAHs) are a group of chemicals with endocrine disruptive potential that are released into the environment through industrial processes, food preparation, and tobacco smoke, with benzo[a]pyrene (BaP) (Figure 6) being one of the most toxic.

In the NANHES 2001–2006, total urinary PAH was analyzed in 3189 participants from 6–19 years of age. The results have shown that urinary PAH concentration was positively correlated with BMI and obesity, with the strongest association in children 6–11 years of age [116]. Another study, conducted in Iran on a smaller number of participants from 6–18 years of age, also showed that urinary monohydroxy PAH was associated with an increased risk of obesity [117]. In addition, a cross-sectional study that used data from NANHES 2003–2008 revealed that simultaneous exposure to PAHs and tobacco smoke synergistically increased the risk of obesity [118]. In other studies, exposure to PAHs was found to be associated with metabolic syndrome, diabetes, and the risk of cardiovascular disease, all health conditions that are strongly connected with obesity [117,119,120,121].

In laboratory studies, PAHs were shown to have a significant impact on adipose tissue functioning both in vitro and in vivo, although the effect of such influence may be quite different. Specifically, the exposure of BaP in combination with PCBs was shown to down-regulate adipogenesis genes while increasing inflammatory gene expression through the up-regulation of AhR signaling in 3T3-L1 cells [122]. These findings corroborate the observation on BaP-induced inhibition of human MSC-derived adipogenesis mediated by AhR activation [123]. Specifically, BaP was shown to down-regulate AhR protein expression and increase AhR translocation to the nucleus, as well as to inhibit PPARγ, resulting in the down-regulation of adipogenesis in canine adipose-derived MSCs [124].

In contrast to in vitro investigations, in vivo studies demonstrated the profound obesogenic effect of PAH exposure. Specifically, prenatal PAH exposure to BALB/cByj mice through the maternal inhalation of PAH mixtures was shown to increase offspring adiposity through an increase in PPARγ and C/EBPα expression, at least partially due to a decrease in PPARγ promotor DNA methylation [125]. It has been also demonstrated that the adipogenic effect of prenatal BaP exposure in mice may be glutathione-dependent [126]. In utero rat exposure to another PAH, 2-aminoanthracene (2AA), was also shown to induce adipogenesis in offspring [127]. BaP was shown to accumulate in murine adipose tissue with a subsequent inhibition of adrenaline-induced lipolysis [128], thus promoting weight gain in C57Bl/6J mice [129].

Prenatal phenanthrene exposure in mice was also shown to impair adipokine levels through the alteration of adiponectin and resistin gene DNA methylation, thus promoting glucose intolerance [130]. Another adipose tissue-targeted mechanism contributing to insulin resistance upon PAH exposure may involve an increase in insulin receptor substrate 2 methylation [131].

It is also notable that obesity may promote 7, 12-dimethylbenz [a] anthracene-induced carcinogenesis both in NIH/3T3 fibroblasts and exposed C57BL/6J female mice [132].

Therefore, the existing findings revealed a significant association between PAH exposure, obesity, and obesity-associated metabolic disturbances. These observations are consistent with in vivo laboratory studies indicating increased adipogenesis through a decrease in PPARγ promotor DNA methylation (Figure 1), lipid overaccumulation due to the inhibition of lipolysis, and insulin resistance (Figure 2). In contrast, in vitro data demonstrate that PAH-induced AhR signaling may result in the inhibition of adipogenesis via the down-regulation of PPARγ. Such an inconsistency may be mediated by the differences in PAH doses used in in vivo and in vitro studies as well as by the mechanisms of PAH metabolism in the organism, thus raising a question on the estimation of conditions for consistency between in vivo and in vitro models.

## 7. Dioxin

**Figure 7:**
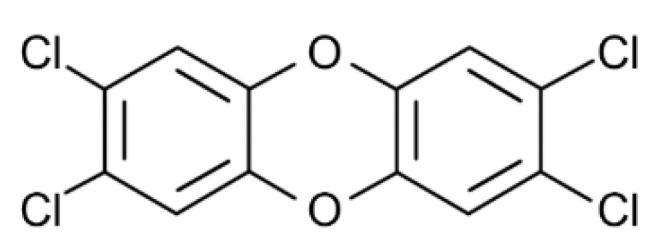
The chemical structure of 2,3,7,8-tetrachlorodibenzo-p-dioxin (TCDD).

The relation between exposure to 2,3,7,8-Tetrachlorodibenzo-p-dioxin (TCDD) (Figure 7), the most prominent member of dioxin and other dioxin-like compounds, to obesity and metabolic effects in general has been examined in several epidemiologic studies producing conflicting data. 

The Seveso Women’s Health Study (SWHS), a cohort study of the health of the women relying on the data obtained after a chemical explosion on 10 July 1976 in Seveso, Italy, prompted Warner et al. [133] to examine the relation of serum TCDD to diabetes, metabolic syndrome, and obesity more than 30 years later. The study enrolled 981 women who were aged from newborns to 40 at the time of the explosion and who were residents of the most contaminated areas. However, the authors did not find association between a 10-fold increase in serum TCDD and obesity. An increased prevalence of metabolic syndrome was observed; however, only among women who were ≤12 years of age at the time of the explosion [133].

In a cross-sectional descriptive study aiming to investigate the link between abdominal obesity and concomitant exposure to serum dioxins (PCDD/Fs), seventeen 2,3,7,8-substituted PCDD/Fs congeners were measured [134]. The authors hypothesized that the subjects with the highest serum PCDD/Fs levels adjusted as TEQDF-1998 and abdominal obesity levels had elevated the chances of developing insulin resistance (IR). In a cross-sectional study evaluating the associations of body burden levels of dioxins and related compounds with the prevalence of metabolic syndrome among the 1374 participants from the general population in Japan, it was found that the body burden levels of dioxins and related compounds (particularly, DL-PCBs) were associated with metabolic syndrome [135]. Furthermore, the same study revealed that high blood pressure, elevated triglycerides, and glucose intolerance were also connected to the levels of these pollutants. In a study investigating 1234 nondiabetic persons living near a deserted pentachlorophenol factory, the association between exposure to dioxins and insulin resistance was investigated [136]. The study proved a positive association between serum dioxins and the prevalence of insulin resistance. The different results obtained by the studies can be partly explained by the fact that the chemical concentration in the blood may be lower in obese subjects due to dilution, while the actual cumulative exposure may be higher due to an extended half-life [137]. 

The role of this class of chemicals in obesity remains to be further elucidated, especially when considering the hydrocarbon receptor (AhR) as the main mediator of dioxins’ toxicity. AhR has been implicated in the regulation of energy metabolism and is currently being investigated as a potential therapeutic target for obesity [138]. Specifically, 2,3,7,8-tetrachlorodibenzo-p-dioxin (TCDD) exposure was shown to inhibit adipogenesis in 3T3-L1 cells through the AhR-dependent down-regulation of PPARγ and C/EBPα at an early stage of differentiation, whereas the dioxin-induced inhibition of glucose uptake was not AhR-dependent [139]. In addition, the inhibitory effect of TCDD on adipogenesis in C3H10T1/2 cells was shown to be aggravated by the epidermal growth factor (EGF) and the fibroblast growth factor (FGF) that were shown to potentiate AhR activation and the subsequent down-regulation of PPARγ expression [140]. Inhibition of PPARγ by dioxin may be at least partially mediated by the down-regulation of PPARγ coactivator 1 alpha production in murine 3T3-L1 adipocytes [141]. ERK activation was also considered as the potential mechanism of the inhibitory effect of TCDD on PPARγ1 expression in C3H10T1/2 cells [142]. In addition, the dioxin-induced inhibition of both isoforms p30 and p42 of C/EBPα may be characterized as an additional mechanism of adipogenesis inhibition in the 3T3-L1 preadipocyte cell line [143].

In addition to the inhibition of PPARγ and C/EBPα in the adipose tissue of exposed guinea pigs, dioxin was also shown to down-regulate sterol regulatory element binding protein (SREBP) 1 and 2, a key regulator of lipid anabolism, as well as its downstream genes, acetyl-CoA carboxylase and HMG-CoA synthase [144]. However, an earlier study demonstrated that TCDD exposure is also capable of inhibiting adipose tissue lipoprotein lipase activity, thus decreasing lipid catabolism [145].

In contrast to in vitro data, in vivo studies have demonstrated a role of TCDD in obesity promotion in laboratory rodents. Specifically, in female C57BL/6J mice fed HFD, TCDD exposure resulted in increased adiposity and reduced adipose tissue triglyceride lipase mRNA expression, as well as elevated liver triglyceride content along with stearoyl-coA desaturase-1 mRNA expression, altogether being associated with higher AhR and androgen receptor mRNA expression [146]. Correspondingly, in another study, TCDD exposure was shown to promote obesity-associated non-alcoholic fatty liver disease in C57BL/6J mice fed a high-fat diet [147].

Moreover, transient low-dose TCDD exposure was shown to induce post-exposure weight gain and glucose intolerance in C57BL/6 mice [148]. However, TCDD-induced hyperglycemia was also shown to be sex-specific, being characteristic only for female C57BL/6 mice [149].

Taken together, the existing data on the role of dioxin exposure in obesity are inconsistent. Despite certain positive findings, other epidemiological studies did not reveal an association between dioxin exposure and obesity. The above-mentioned laboratory data indicate that dioxin-induced AhR signaling may be responsible for the inhibition of adipogenesis in vitro, whereas in vivo studies demonstrate that dioxin exposure may promote excessive adiposity and obesity-associated metabolic disturbances in laboratory animals. Hypothetically, the AhR-dependent down-regulation of adipogenesis upon dioxin exposure may result in adipose tissue dysfunction and increased ectopic lipid accumulation, thus promoting additional metabolic risk, although this suggestion requires further elaboration.

## 8. Bisphenol A (BPA)

**Figure 8:**
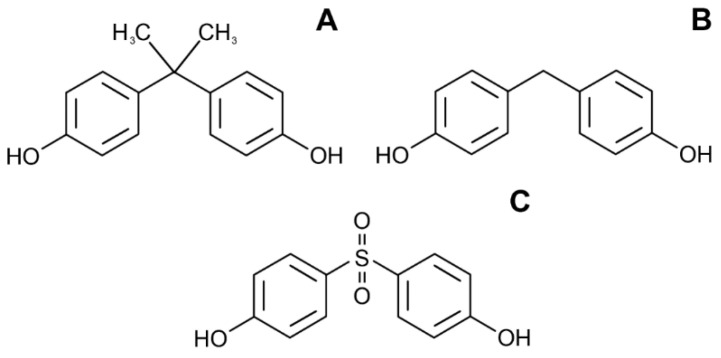
The structure of bisphenol A (**A**) and related compounds bisphenol F (**B**) and A (**C**).

Bisphenol A (BPA) (Figure 8A) is a synthetic organic chemical with endocrine disrupting potential that is used with other chemicals in the production of various plastic products and epoxy resins [150]. 

Several recent studies have demonstrated the link between BPA and obesity development. In 296 women of reproductive age, BPA urinary levels were positively corelated with BMI and waist circumference [151]. In the recently published and previously mentioned Korean National Environmental Health Survey (KoNEHS) 2015–2017, higher BPA urinary levels showed a significantly higher risk of developing diabetes mellitus and obesity [152]. In the Korean cross-sectional study (*n* = 702), urinary BPA concentration was associated with abdominal obesity in women but not in men, with the strongest association determined in postmenopausal women, indicating the roles of gender and women’s menopausal status [153]. The data processed from the U.S. NANHES (2003–2006) study on 2747 adult subjects showed that participants in the highest BPA urinary quartiles had a higher obesity incidence when compared to the lowest BPA quartile, and generally higher BPA levels were associated with higher body weight and central obesity [154]. Growing evidence suggests that BPA can interfere with physiological metabolism and promote obesity in children and adolescents [155]. In a study investigating 1326 children and adolescents, it was observed that in male participants, BPA urinary levels were not linked to obesity. However, in females, this association was found, especially among girls entering the pubertal stage (BPA > 2 mg/L), and their risk of being overweight was doubled when compared to those with lower urine BPA levels [156]. A large study in Chinese school children (more than 2000 participants from middle and high school) demonstrated that urine BPA levels were positively associated with BMI [157]. The limitation of the most presented studies is their cross-sectional design, so there is a need for longitudinal studies to further scrutinize the link between BPA and obesity development. 

Recent studies demonstrate that BPA exposure may significantly affect adipogenesis through the modulation of key adipogenesis regulators (PPARγ and C/EBPs) through interference with receptor signaling. Specifically, BPA exposure was shown to up-regulate adipogenesis in human adipose stromal/stem cells due to the activation of PPARγ and C/EBPα in an estrogen receptor-dependent manner [158]. It has also been shown that the adipogenic effect of BPA may be mediated through its interference with glucocorticoid signaling. Specifically, BPA exposure was shown to stimulate PPAR-γ mRNA expression in human visceral (pre)adipocytes by increasing mRNA expression and the activity of 11β-HSD1, an enzyme involved in cortisol formation [159]. In addition to the earlier reported role of PPARγ and C/EBPα, the adipogenic effect of BPA exposure in 3T3-L1 cells may be also mediated by the increase in transcriptional activity of the glucocorticoid receptor and C/EBPδ [160]. At the same time, another study demonstrated that BPA-induced adipogenesis in human preadipocytes is dependent on estrogen receptor signaling, rather than the glucocorticoid pathway [161]. It is also notable that the impact of BPA on PPARγ signaling during adipogenesis in committed 3T3L1 and uncommitted NIH3T3 preadipocytes may be mediated by the BPA-induced reduction of preadipocyte peroxisome proliferator-activated receptor gamma (PPARγ) promoter methylation [162]. The activation of the PI3K/Akt pathway is also considered as the potential mechanism of BPA proadipogenic activity in 3T3-L1 cells [163]. 

In addition to the modulation of adipocyte proliferation and differentiation, BPA exposure promoted lipid accumulation, proinflammatory cytokine production, and reduced insulin sensitivity in mature 3T3-L1 adipocytes [164]. Moreover, BPA-induced insulin resistance in adipocytes was shown to be independent of adipogenesis, being associated with reduced insulin-induced Akt phosphorylation and increased proinflammatory cytokine mRNA levels and being indicative of the role of BPA-induced inflammation in insulin resistance [165]. Correspondingly, the BPA-induced alteration of insulin receptor phosphorylation and signaling in adipocytes derived from subcutaneous adipose tissue and differentiated 3T3-L1 cells was shown to be JNK-dependent [166]. Moreover, environmentally relevant doses of BPA were also shown to decrease adiponectin production by human adipose tissue [167], which may significantly contribute to insulin resistance.

Being in agreement with in vitro studies, in vivo experiments also demonstrated the impact of BPA exposure on adipogenesis. Specifically, exposure to low doses, but not high doses, of BPA induced a significant increase in adipose tissue mass with an elevation in both adipocyte size and volume, as well as circulating leptin levels and insulin resistance [168]. Perinatal BPA exposure in Sprague-Dawley rats was shown to increase white adipose tissue mass through inducing adipocyte hypertrophy due to the up-regulation of PPAR-γ, C/EBP-α, SREBP-1C, LPL, FAS, and stearoyl-CoA desaturase 1 (SCD-1), although this effect was observed only in females [169].

At the same time, in vivo studies revealed the significant sex-specific effects of BPA on adiposity [170]. Specifically, BPA exposure was shown to induce an increase in body adiposity and adipose tissue inflammation in STD-fed female C57BL/6J mice, whereas no effect was observed in HFD-fed and/or male mice [171]. In turn, another study demonstrated a significant BPA-induced increase in the body adiposity in male Sprague–Dawley rats [172]. Given this inconsistency and the observation of sex-specific effects, it is proposed that BPA is not considered as a specific obesogen in laboratory rodents (C57BL/6JxFVB mice), although it programs for metabolic dysregulation [173].

Several studies have evaluated the impact of BPA derivatives BPA-glucuronide (BPA-G) [174] and bisphenol A diglycidyl ether (BPA-DGE) [175] on adipogenesis. Specifically, BPA-glucuronide (BPA-G), the main BPA metabolite, was also shown to induce adipogenesis in human and 3T3L1 murine preadipocytes through a mechanism involving estrogen receptor activation without the direct estrogenic activity of the compound [174]. Bisphenol A diglycidyl ether also possessed a more profound adipogenic effect as compared to BPA by inducing adipogenesis both in mesenchymal stromal stem cells and 3T3-L1 preadipocytes, whereas BPA was capable of inducing an adipogenic response only in the latter [175]. 

A comparative analysis of various bisphenol species using the preadipocytic 3T3-L1 cell line demonstrated that bisphenol F and especially bisphenol S possess a more profound adipogenic effect as compared to BPA, as demonstrated by higher PPARγ and C/EBPα protein expression [176]. A similar PPARγ-mediated effect was observed in primary human preadipocytes [177]. Both BPA and BPS were shown to activate PPARγ in murine preadipocytes through targeting the PPARγ response element, although the mechanism may be quite different [178]. Correspondingly, BPA and BPS induced distinct transcriptional patterns in differentiating human primary preadipocytes that may underlie the differences in adipogenic effects of these compounds. While BPA was shown to affect mainly the pathways involved in liver X receptor/retinoid X receptor (LXR/RXR) activation, hepatic fibrosis, cholestasis, and atherosclerosis signaling, BPS exposure perturbed adenosine monophosphate-activated protein kinase (AMPK) signaling, cholesterol biosynthesis, and adipogenesis pathways, as well as LXR/RXR and PPARα/RXRα activation [179]. In agreement with in vitro observations, in vivo prenatal BPS exposure was shown to result in white adipose tissue hypertrophy and the up-regulation of PPARγ gene expression in HFD-fed mice, being indicative of the role of BPS in increasing susceptibility to dietary obesity [180].

Certain studies also demonstrated that bisphenol AF may also promote adipogenesis and induce proinflammatory signaling pathways in murine 3T3L1 preadipocytes [181]. 

Generally, BPA exposure may be considered a risk factor for human obesity as evidenced by epidemiological studies. The promotion of PPARγ and C/EBPα-dependent adipogenesis upon exposure to BPA may be mediated by glucocorticoid and estrogen receptor signaling, also involving the up-regulation of C/EBPδ (Figure 2). The epigenetic effects of BPA as well as the induction of adipose tissue inflammation may also contribute to obesity pathogenesis and obesity-associated insulin resistance (Figure 5). Involvement of the estrogen receptor may also underlie certain sex-specific obesogenic effects of BPA. It is also notable that other bisphenol (S, F, AF) species may possess obesogenic effects through similar mechanisms.

## 9. Phthalates

**Figure 9:**
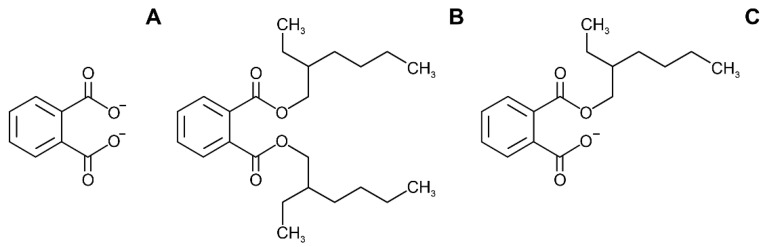
The common structure of phthalates (**A**), as well as the most common representatives di(2-ethylhexyl)phthalate (DEHP) (**B**) and its metabolite mono(2-ethylhexyl)phthalate (MEHP) (**C**).

Phthalates (Figure 9), diesters of 1,2-benzendicarboxylic acid, are a group of chemicals used as additives in plastics. These chemicals are listed as endocrine disrupting chemicals [182]. Many epidemiological studies have examined the association between phthalates (measured as metabolites in urine) and body weight and obesity [183,184,185,186,187]. Results from a cross sectional study conducted on 242 participants (6–18 years of age) have shown an association between urinary phthalate metabolites and obesity, triglyceride, and blood pressure [183]. Hatch et al. used data from the US National Health and Nutrition Examination Surveys (NANHES) and revealed a number of different positive associations between phthalate exposure and BMI and waist circumference, with the most coherent results in males from 20–59 years old [184]. A study investigating 128 newborns demonstrated the positive association between urine di(2-ethylhexyl)phthalate (DEHP) levels and an increase in body weight at the 3rd month of life [185]. In the Korean National Environmental Health Survey (KoNEHS) conducted between 2015 and 2017 with a sample size of 3782 subjects, DEHP and benzylbutylphthalate (BzBP) urine levels were found to be associated with obesity [152]. A case–control study performed in Iran (*n* = 320) showed a positive association between phthalates metabolites and the BMI of participants [186]. Contrary to this, a Korean cross-sectional study of 702 participants did not find a significant association between the urinary levels of six phthalates metabolites and general and/or abdominal obesity in both genders [153]. And lastly, a negative association between phthalates metabolites and BMI was reported in a study investigating these parameters in American children [187]. It is evident that there are controversial data describing the connection between exposure to phthalates and obesity. It should be highlighted that most of the studies are of cross-sectional design. Hence, there is a need for large prospective studies which would confirm or infirm the existence of this connection.

The existing data demonstrate the adipogenic potential of phthalate exposure due to the up-regulation of PPARγ [188]. Specifically, the role of PPARγ activation in the adipogenic response in 3T3L1 cells was demonstrated for MEHP and DEHP [189], as well as other phthalates, including monobenzyl phthalate (MBzP) and mono-sec-butyl phthalate (MBuP) [190], benzyl butyl phthalate (BBP) [191,192], and diisononyl phthalate (DINP) [193]. It is also notable that the DEHP-induced up-regulation of PPARγ also requires the activation of Med1 and PGC-1α coregulators in 3T3L1 cells [189].

Correspondingly, certain studies demonstrate that phthalate exposure to pluripotent bone marrow stromal cells may promote adipogenesis at the expense of osteoblastogenesis [194] and Leydig cell differentiation [195] through the up-regulation of PPARγ and C/EBPα expression.

Phthalate-induced adipogenesis in 3T3L1 cells may be also dependent on the activation of the glucocorticoid receptor [196]. In agreement, molecular docking analysis demonstrated that dicyclohexyl phthalate (DCHP) and mono-cyclohexyl phthalate (MCHP) bind GR active sites with biding affinities close to that of dexamethasone [197].

In agreement with the phthalate-induced modulation of adipogenesis, exposure to this compound was also shown to affect adipokine secretion. Specifically, a DEHP-induced increase in leptin and FABP4 mRNA expression was associated with adiponectin down-regulation [198]. In contrast, another study revealed a significant inhibition in adipokine production in 3T3-L1 adipocytes upon MEHP exposure [199]. 

In parallel with the promotion of 3T3-L1 murine preadipocyte differentiation to adipocytes, MEHP exposure also increased the expression of genes implicated in lipid uptake, biosynthesis, and accumulation [200]. In addition, MEHP-induced lipid accumulation in 3T3-L1 adipocytes may be associated with the up-regulation of the Notch pathway as evidenced by increased Notch-1 and Jagged-2 expression [201]. The activation of the TYK2/STAT-3 pathway may be also considered as another candidate mechanism promoting 3T3-L1 adipocyte differentiation and lipid accumulation [202]. 

In contrast, one of the studies demonstrated that DEHP exposure may induce white adipocyte browning, as evidenced by the increased expression of *PPARG*, *ADRB1*, *ADRB3*, *PPARGC1a*, and *UCP1* [203]. MEHP accumulation in adipocytes was also shown to reduce adipocyte size through a significant increase in lipolysis, glucose uptake, glycolysis, mitochondrial respiration, and mitochondrial biogenesis in 3T3-L1 cells [204]. 

The results of in vivo studies generally correspond to in vitro data, demonstrating the impact of phthalate exposure on body adiposity and obesity-associated metabolic disturbances. Specifically, i.p. DEHP injection to C57BL/6J mice resulted in a significant increase in adipose tissue weight, as well as circulating total cholesterol, glucose, and triglyceride levels [205]. A similar effect was observed following in utero phthalate exposure. In utero MEHP exposure significantly increased body weight and adipose tissue mass along with the development of hyperglycemia and dyslipidemia in mouse offspring [206]. In agreement, in utero exposure to DEHP in C57BL/6J mice was shown to increase body adiposity in the offspring accompanied by the elevation of circulating leptin, insulin, lipid, and glucose levels [207], as well as blood pressure through the dysregulation of AT1R signaling and eNOS activation [208], thus promoting metabolic syndrome. 

The observed phthalate-induced insulin resistance in male Wistar rats may be associated with a decrease in adipocyte glucose uptake, resulting in hyperglycemia and insulin resistance in rats through the down-regulation of insulin receptor and IRS-1 mRNA expression, as well as the inhibition of Akt phosphorylation [209]. Moreover, the protective role of ascorbic acid and α-tocopherol demonstrates the role of oxidative stress in the alteration of adipose tissue insulin signaling [209]. 

BBP exposure in C57BL/6 mice was also shown to potentiate the adipogenic effect of HFD through an increase in liver and adipose tissue mass, also inducing insulin resistance, although the effect was observed only in moderate, but not low or high, doses [210]. 

DEHP exposure was also shown to induce rat adipose tissue infiltration with macrophages with the subsequent secretion of TNFα and IL-1b, which promoted adipose tissue dysfunction and altered lipid metabolism [211]. In agreement, in utero DEHP exposure in Sprague–Dawley rats induced both adipose tissue and systemic inflammation in parallel with increasing preadipocyte differentiation [212]. It is also proposed that PPARγ may be at least partially responsible for the proinflammatory response in differentiated murine adipocytes in parallel with the adipogenic effects [213]. 

Additional mechanisms linking phthalate exposure and obesity pathogenesis were also proposed. Specifically, the epigenetic effects of phthalate exposure were shown to contribute to phthalate-induced adipogenesis in MSCs and high-fat fed rats [214,215], although this effect in rats is expected to be sex-specific [216]. In addition, the most recent study also demonstrated that altered gut microbiota with a decrease of the Firmicutes-to-Bacteroidetes ratio may be associated with DEHP-induced obesity in mice [217].

Despite the contradictory epidemiological data, laboratory findings demonstrate that exposure to phthalates promoted adipogenesis through the up-regulation of C/EBPα and PPARγ signaling (Figure 2), as well as lipid accumulation in adipocytes due to the activation of lipid biosynthesis. In addition, phthalate-induced adipokine dysregulation, adipose tissue inflammation, and epigenetic effects may also contribute to obesity and obesity-associated metabolic disturbances.

## 10. Diethylstilbestrol (DES)

**Figure 10:**
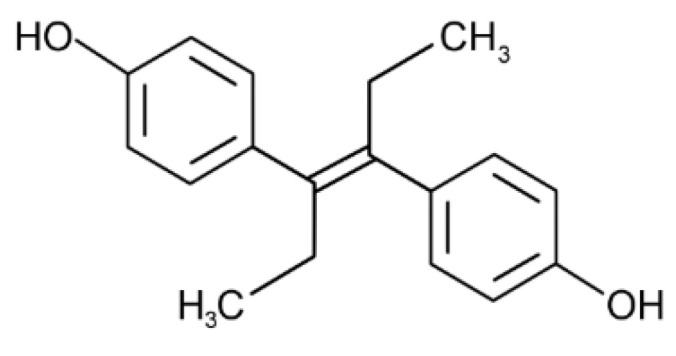
The structural formula of diethylstilbestrol (DES).

Diethylstilbestrol (DES) (Figure 10) is a synthetic drug with estrogen activity that was used as a therapy for the prevention of miscarriage and other pregnancy complications during the period from 1940–1970 and was recognized as a potential obesogenic in animal studies [218,219]. However, there are only a few epidemiological studies that examined the obesogenic potential of DES in in utero-exposed children. A large prospective study, covering three cohorts, has shown that DES-exposed women had a slightly greater weight than unexposed controls [220]. Another study performed using data from the Collaborative Perinatal Project (*n* = 34,419), found strong a positive association between prenatal exposure to DES at 3–4 months and 4–5 months of pregnancy and obesity in children at 7 years of age [221]. 

DES exposure was shown to induce adipogenesis via the estrogen receptor-mediated activation of PPARγ and C/EBPα expression in 3T3-L1 preadipocytes with the subsequent up-regulation of target genes including aP2, FAS, and LPL. A similar effect was observed in vivo [218]. In contrast, in mature adipocytes, DES exposure resulted in a significant decrease in PPAR expression due to the down-regulation of ERα and ERβ [222]. 

Certain studies demonstrated that DES may also affect other mechanisms implicated in obesity pathogenesis. Specifically, DES was capable of inhibiting mitochondrial respiration and glycolysis as well as activating ERK in 3T3-L1 adipocytes in an ER-dependent manner [223]. In addition, DES exposure was shown to induce WAT inflammation in obese mice [224]. 

Taken together, epidemiological data on the potential role of DES as an environmental obesogen are insufficient, although laboratory findings indicate that adipose tissue could be considered as a potential target for this pollutant.

## 11. POPs Mixtures and Obesity

In view of the recent rate of environmental pollution, humankind is more frequently exposed to a mixture of various chemicals rather than to one particular pollutant [225]. Therefore, an investigation of the impact of various POPs mixtures on obesity and adipogenesis is of particular interest. 

Data from the Center for Health Assessment of Mothers and Children of Salinas (CHAMACOS) study demonstrated that obesity is directly associated with serum dichlorodiphenyltrichloroethane, hexachlorocyclohexane, and PBDE-47 concentrations, whereas PBDE-153 was characterized by an inverse association [93]. Another study also revealed an association between obesity and urinary mono (carboxyoctyl) phthalate, BPA, and BPS levels in the NHANES 2013–2014 cohort [226]. Although these findings clearly demonstrate that exposure to POPs mixtures may be associated with anthropometric markers of obesity, the particular effect of multiple pollutant co-exposure is unclear.

Only single studies demonstrated the impact of POPs mixtures on obesity and adipogenesis mechanisms. Specifically, the most recent study demonstrated that a mixture of POPs in human exposure-relevant doses promoted adipogenesis in 3T3-L1 cells, inducing higher lipid accumulation as compared to single chemicals [53]. It has been also demonstrated that female zebrafish exposed to natural mixture of POPs are characterized by significantly increased body weight, which may be associated with the modulation of PPARγ and C/EBPa signaling or with other endocrine disturbances [227]. Further in vivo and in vitro studies aimed at the investigation of the interactive effects of POPs on adipogenesis upon co-exposure are required [228].

## 12. A Summary of Key Targets for POPs Obesogenic Effects

The reviewed studies demonstrated that the obesogenic effects of POPs may be mediated by their impact on adipogenic transcription regulators, namely PPARγ and C/EBPs, as well as nuclear receptors including ER and GR. The role of these mechanisms in adipogenesis and adipose tissue functioning will be briefly reviewed. 

### 12.1. PPAR and C/EBP

The above reviewed studies clearly indicate that PPARγ may be considered as a target for all discussed POPs. PPARγ is a key regulator of adipogenesis that activates preadipocyte differentiation and the expression of adipocyte-specific genes involved in lipid metabolism and accumulation, insulin sensitivity, and thermogenesis [229]. Correspondingly, targeted adipose tissue PPARγ knockout results in reduced adipogenesis [230]. It is also notable that PPARγ is also essential for the survival of mature adipocytes [231]. At the same time, whole-body PPARγ knockout was shown to be lethal due to placental defects [232]. 

PPARγ expression is regulated by a wide spectrum of transcription factors, with the C/EBP family playing a key role [233]. C/EBPβ and C/EBPδ are induced at early steps of adipogenesis, subsequently up-regulating PPARγ expression [234]. PPARγ activity is integrated with another transcription factor, C/EBPα, and both transcriptions potentiate the expression of each other [234]. However, certain studies demonstrated that C/EBPα is unable to induce adipogenesis without PPARγ, which is considered as the proximal regulator of adipogenesis [235].

PPAR activation is also regulated by epigenetic modifications including chromatin remodeling and histone acetylation and methylation [236]. Specifically, the demethylation of PPARγ promotor during adipogenesis is associated with its activation [237]; this was also observed upon exposure to POPs.

PPAR activation is also known to be involved in the regulation of insulin sensitivity through the up-regulation of GluT4, PI3K, and IRS1/2 [238]. However, upon PPARγ overexpression, increased glucose and fatty acid uptake, in parallel with increased adipogenesis, may promote weight gain, resulting in visceral obesity [229]. In view of the data demonstrating the interference of POPs with insulin signaling through the inhibition of insulin receptor IRS1/2 and Akt phosphorylation, as well as PTEN activation (Figure 5), the insulin-sensitizing effect of PPARγ activation may be abrogated by exposure to POPs. 

Therefore, aberrant PPARγ activation upon exposure to POPs may be considered as one of the key mechanisms mediating the obesogenic effects of POPs, although the particular mode of POP-PPAR interaction is still to be further explored [239].

### 12.2. Estrogen Receptor (ER)

Certain POPs, including DDT, PCB, BPA, and DES, were shown to mediate their adipogenic effects through targeting estrogen receptors. These observations correspond to the earlier reported estrogenic activity of PCBs and DES. 

ERα and ERβ are known to be involved in the regulation of adipose tissue functioning as well as adipocyte differentiation, although the effects are receptor-specific [240]. 

Erβ was shown to inhibit PPARγ transcription and activity resulting in reduced adipogenesis [241], which may be at least partially mediated by the competition between PPARγ and Erβ for coactivator binding [242]. Correspondingly, selective ERβ agonists were shown to inhibit basal and PGC-1-coactivated PPAR-γ transactivation [243]. In vivo, ERβ activation significantly reduced both visceral and subcutaneous adipose tissue mass in HFD-fed mice [244]. In turn, Erβ inhibition is associated with the activation of PPARγ signaling and subsequent adipogenesis [245].

At the same time, ERα is believed to have a predominant role in the regulation of adipogenesis when compared to Erβ. Erα activation upon estradiol treatment was shown to increase 3T3-L1 adipocyte differentiation through C/EBPα signaling, along with increased GluT4 expression and improved insulin sensitivity [246]. Correspondingly, Erα knockdown is associated with adipocyte hypertrophy [247].

Erα expression in white adipocytes is also associated with reduced visceral adiposity through the modulation of mitochondrial dynamics due to the up-regulation of Polg1 (mtDNA polymerase γ-subunit) [248]. Correspondingly, ERα activation is also associated with adipocyte beiging through the up-regulation of AMPK, adipose tissue triglyceride lipase (ATGL), and UCP-1 expression [249].

Based on the above mentioned studies, it is considered that physiologically high Era expression is required for adipogenesis and normal adipose tissue functioning, whereas its Era down-regulation affects adipogenesis and promotes adipocyte hypertrophy together with ectopic lipid accumulation [250].

At the same time, certain studies demonstrated that Era signaling may also repress adipogenesis through the up-regulation of AKT with the subsequent recruitment of co-repressors GATA3 and β-catenin/TCF4 complex to the PPARγ promoter, ultimately leading to reduced PPARγ activity. Moreover, AKT activation was also shown to facilitate ERα nuclear translocation and localization at the PPARγ gene promoter through the inhibition of trimethylation of lysine 27 of histone H3 (H3K27me3) and increased wingless-integrated (Wnt) 1and 10b expression [251]. Correspondingly, the role of H3K27me2 and H3K27me3 in Era induced a decrease in PPARγ and C/EBPα expression [252].

In agreement with the distinct effects of ERs in adipocytes, it has been demonstrated that the ERα/Erβ ratio is associated with obesity and leptin production, with higher BMI values in cases with a shift to the prevalence of Erβ [253].

The specific patterns of estrogen receptor signaling may mediate the observed sexual dimorphism in the obesogenic response to POPs. Specifically, it has been demonstrated that 17β-Estradiol is capable of inducing Erα and ERβ mRNA expression in female subcutaneous adipocytes, whereas in male cells, only Erα was up-regulated [246]. In addition, Erα deficiency in adipose tissue was shown to induce adipocyte hypertrophy as well as fibrosis and inflammation, being more profound in males [247]. 

Given the significant role of estrogens in the regulation of adipose tissue functioning, the difference in estrogen production and the distinct patterns of ERs distribution in men and women may mediate the observed sex-specific effects of exposure to POPs [254]. 

### 12.3. Glucocorticoid Receptor

The activation of glucocorticoid receptor signaling by its ligand, dexamethasone, is known to be involved in the activation of adipogenesis through the up-regulation of proadipogenic transcription factors [255]. Specifically, GR activation was shown to be involved in the up-regulation of C/EBPβ [256,257] and C/EBPδ [258] expression with the subsequent induction of C/EBPα. It has been also demonstrated that GR-induced adipogenesis may be mediated by KLF-15-induced PPARγ activation [259].

At the same time, certain studies demonstrated that GR signaling is not required for in vivo adipogenesis [260]. Correspondingly, another study demonstrated that the activation of GR signaling by dexamethasone accelerates adipocyte differentiation through the up-regulation of C/EBPα, C/EBPβ, C/EBPδ, KLF5, KLF9, and PPARγ expression, being yet dispensable for adipogenesis, as demonstrated in GR-deficient preadipocytes that were capable of further differentiation [261]. Being in line with these observations, an in vivo study demonstrated that targeted adipocyte GR deletion does not reduce adipose tissue weight and distribution, although it does promote adipose tissue inflammation upon high-fat feeding [262].

At the same time, the particular effect of GR signaling in POPs-associated obesity is unclear, especially in view of the earlier demonstrated predominant role of ER activation upon exposure to POPs [161]. 

## 13. Conclusions

The existing epidemiological data demonstrate a significant association between lifetime and prenatal exposure to POPs and obesity and obesity-associated metabolic disturbances (e.g., type 2 diabetes mellitus and metabolic syndrome), although the existing data are rather insufficient and sometimes contradictory. 

Laboratory in vitro data have clearly and consistently demonstrated the potential mechanisms associated with the interference of exposure to POPs with obesity, including: (i) the effects on adipogenesis regulators (PPARγ, C/EBPα); (ii) the endocrine-disrupting effects and binding to nuclear receptors (ER, GR); (iii) the epigenetic effects; (iv) proinflammatory activity; and (v) the induction of insulin resistance. The involvement of estrogen-like effects of certain POPs and ER signaling may also underlie the sex-specific differences in POPs’ adipogenic effects. Although in vivo data are generally corroborative of the in vitro results, studies in living organisms demonstrated that the impact of POPs on adipogenesis is largely affected by biological factors including sex, age, and period of exposure. 

Therefore, both laboratory and epidemiological data underline the significant role of POPs as environmental obesogens. However, further studies are required to better characterize both the mechanisms and the dose-response effects of exposure to POPs in the etiology of obesity and other metabolic diseases.
